# PromptSMILES: prompting for scaffold decoration and fragment linking in chemical language models

**DOI:** 10.1186/s13321-024-00866-5

**Published:** 2024-07-04

**Authors:** Morgan Thomas, Mazen Ahmad, Gary Tresadern, Gianni de Fabritiis

**Affiliations:** 1 Computational Science Laboratory, Universitat Pompeu Fabra, Barcelona Biomedical Research Park (PRBB), C Dr. Aguiader 88, 08003 Barcelona, Spain; 2 In Silico Discovery, Janssen Pharmaceutica N. V., Turnhoutseweg 30, 2340 Beerse, Belgium; 3 Acellera Labs, C Dr. Trueta 183, 08005 Barcelona, Spain; 4 Institució Catalana de Recerca i Estudis Avançats (ICREA), Passeig Lluis Companys 23, 08010 Barcelona, Spain

**Keywords:** Chemical language models, Scaffold hopping, Scaffold decoration, Fragment linking, Reinforcement learning, De novo molecule generation, Artificial intelligence, Drug design

## Abstract

SMILES-based generative models are amongst the most robust and successful recent methods used to augment drug design. They are typically used for complete de novo generation, however, scaffold decoration and fragment linking applications are sometimes desirable which requires a different grammar, architecture, training dataset and therefore, re-training of a new model. In this work, we describe a simple procedure to conduct constrained molecule generation with a SMILES-based generative model to extend applicability to scaffold decoration and fragment linking by providing SMILES prompts, without the need for re-training. In combination with reinforcement learning, we show that pre-trained, decoder-only models adapt to these applications quickly and can further optimize molecule generation towards a specified objective. We compare the performance of this approach to a variety of orthogonal approaches and show that performance is comparable or better. For convenience, we provide an easy-to-use python package to facilitate model sampling which can be found on GitHub and the Python Package Index.

**Scientific contribution**

This novel method extends an autoregressive chemical language model to scaffold decoration and fragment linking scenarios. This doesn’t require re-training, the use of a bespoke grammar, or curation of a custom dataset, as commonly required by other approaches.

## Introduction

The drug design process is a multi-stage process. Particularly in later drug design stages, it is desirable to fix a molecule to a known core sub-structure or “scaffold” and explore different decoration groups. Or alternatively, to fix certain periphery sub-structures or “fragments” and explore chemical sub-structures to combine them together, known as fragment linking, or scaffold hopping. Such chemically constrained modification has useful applications, for example, to tweak molecular properties of a lead series, to combine two weakly binding fragments identified through fragment-based campaigns [1], to optimise proteolysis targeting chimeras that link two warheads [2], or to quickly identify novel intellectual property [3].

Chemical language models (CLMs) are now a well-established method for de novo molecule generation. Seminal methods evidenced their ability to learn to generate valid and novel SMILES strings when trained on a corpus of example SMILES [4], use transfer learning (a.k.a. fine-tuning) to condition generation to a particular chemical sub-space [4, 5], or reinforcement learning (RL) to condition generation in order to maximize an arbitrary objective according to a scoring function(s) that evaluates de novo chemistry [6, 7]. Since seminal works, there has been much research and proposed improvements, such as using diversity filters to penalize excessive exploitation [8], use of experience replay [9] (and augmented variations [10, 11]), or exploration [12] and modification [13, 14] of RL algorithms to accelerate learning. In light of alternative and more complex model architectures, CLMs remain either 1^*st*^ or 2^*nd*^ most performant according to a variety of benchmarks [15–18] and are the most commonly published deep learning model for de novo molecule generation [19]. Furthermore, they have undergone experimental validation evidencing their ability to generate bioactive molecules de novo in the context of drug design [20–25]. Overall CLMs are of increasing utility and importance to augmenting and automating the drug design process.

CLMs based on SMILES notation have also been adapted to these practical requirements of the drug design process. For example, Langevin et al. [26] proposed SAMOA that enforced free or constrained sampling from a CLM to conduct scaffold decoration or two-fragment linking i.e., allowing free sampling at a desired decoration or linking attachment point. Alternatively, Arús-Pous et al. [27] proposed an RNN encoder-decoder architecture regarding the task as a sequence-to-sequence translation, for example, translating an input scaffold to the output predicted decorations. This differs from the decoder-only CLMs discussed until this point and requires slicing of a molecular dataset into scaffolds and decorating groups before training the encoder-decoder model. This architecture was used in LibINVENT [28] to generate scaffold-constrained de novo molecules adhering to chemical reaction rules by bespoke dataset slicing via handcrafted reaction rules and reaction filter RL objectives. Succeeding this, the architecture was used in LinkINVENT [29] by instead training the encoder-decoder model to translate a pair of molecular fragments to a linker effectively reversing the translation task. Similarly, SyntaLinker [30] uses a conditional transformer model to translate fragments to fully linked molecules. This approach is impractical as it forces training many different CLMs architectures for each task.

Beyond CLMs, graph neural networks have been proposed specifically for the purpose of being able to conduct both unconstrained and scaffold constrained generation with MoLeR [31]. New language-based molecular representations [32] have also been proposed to offer greater flexibility in conducting constrained molecule generation. Further exemplifying the importance and utility of flexibly conducting constrained or unconstrained molecule generation with the same model for drug design.

In this work, we take inspiration from the work of SAMOA and the use of prompts to condition language generation as in models like GPT [33]. Prompts are sequences or partial sequences of a language used to inform future sequence generation. More specifically in this case, we propose to leverage the implicitly learned relationship between molecular sub-structures (as groups of SMILES tokens) by a simple unidirectional decoder-only model when trained on a corpus of molecules. This can be achieved by providing a molecular sub-structure as a prompt at inference time to condition further molecule generation (as well as, constrain the final molecule to contain this sub-structure). Further, the CLM can adapt to this task and optimize for different objectives when combined with RL. In the case of scaffold decoration of more than one attachment point, prompted generation is repeated for each attachment point (integrating any previously generated de novo decorations at each iteration). In the case of fragment linking, one fragment is chosen as the prompt and further fragments are inserted into the de novo generated sequence and evaluated by the CLM.

In contrast to previous work, we demonstrate that scaffold decoration and fragment linking (or scaffold hopping) can be achieved via prompts and RL with available decoder-only models. This avoids the need to design new grammars [32], implement and train encoder-decoder models specific to the task [28, 29], or even design bespoke model architectures [31]. Note that simple omitting of this approach results in plain de novo generation with the CLM. In comparison to the most similar approach SAMOA, we manipulate the SMILES string and re-introduce it to the CLM iteratively such that the CLM observes all of the molecular structure at each iteration to inform conditional generation.

## Methods

Autoregressive language-based models learn the probability of the next token in a sequence conditional upon previously observed tokens. Therefore, de novo string generation can be conducted by supplying a start token (e.g., “GO”) and predicting the probability over the next token in the vocabulary, sampling from that probability distribution to select the next token (e.g., “C”), and then using the sampled token as the input in the subsequent iteration. This is repeated until a stop token (e.g., “EOS”) is sampled indicating the termination of generation. However, an initial sequence of tokens can be provided from a user-specified prompt upon which further generation is conditional, as is the case with large-language models such as GPT [33].

The SMILES grammar [34] is an interpretable string notation of the 2D graph traversal of a molecule (and can include stereochemistry). Therefore, a CLM trained on the SMILES grammar can conduct conditional (a.k.a. constrained from a chemistry perspective) generation by completion of a SMILES prompt, for example, a benzene prompt (“[GO]c1ccccc1”) could be continued into benzanoic acid (“[GO]c1ccccc1C(=O)O[EOS]”). However, there are two obvious limitations to this from the chemistry perspective (1) string extension means that atoms are added to the last atom in the SMILES string and therefore the last atom must be the desired attachment point, and (2) string generation is unidirectional, atoms can only be added to one single attachment point. Conveniently, a single molecule has multiple SMILES representations that can be controlled depending on the starting point and algorithm used for graph traversal. Therefore, SMILES can be generated such that the attachment point of interest can be re-arranged to be the last atom in the SMILES string. Given this simple observation, prompt-based SMILES generation can be repeated for multiple attachment points. This also results in conditional generation based on previously extended attachment points. However, this iterative approach to prompted SMILES generation introduces a new limitation: token generation continues until a stop token has a high probability and is therefore sampled, i.e., the model is likely to consider the molecule complete after the first iteration. Therefore, we propose that RL will fine-tune the model to adapt to the new task of iterative prompt-based generation.

To demonstrate this approach, we use a recurrent neural network (RNN) architecture as the CLM in combination with the reinforcement learning (RL) strategy proposed by REINVENT, for comparative purposes. We endeavoured to follow the guidelines outlined by the baseline method for each experiment with regard to RNN architecture and training, for further detail see Appendix A.

### Prompt-based scaffold decoration

**Fig. 1:**
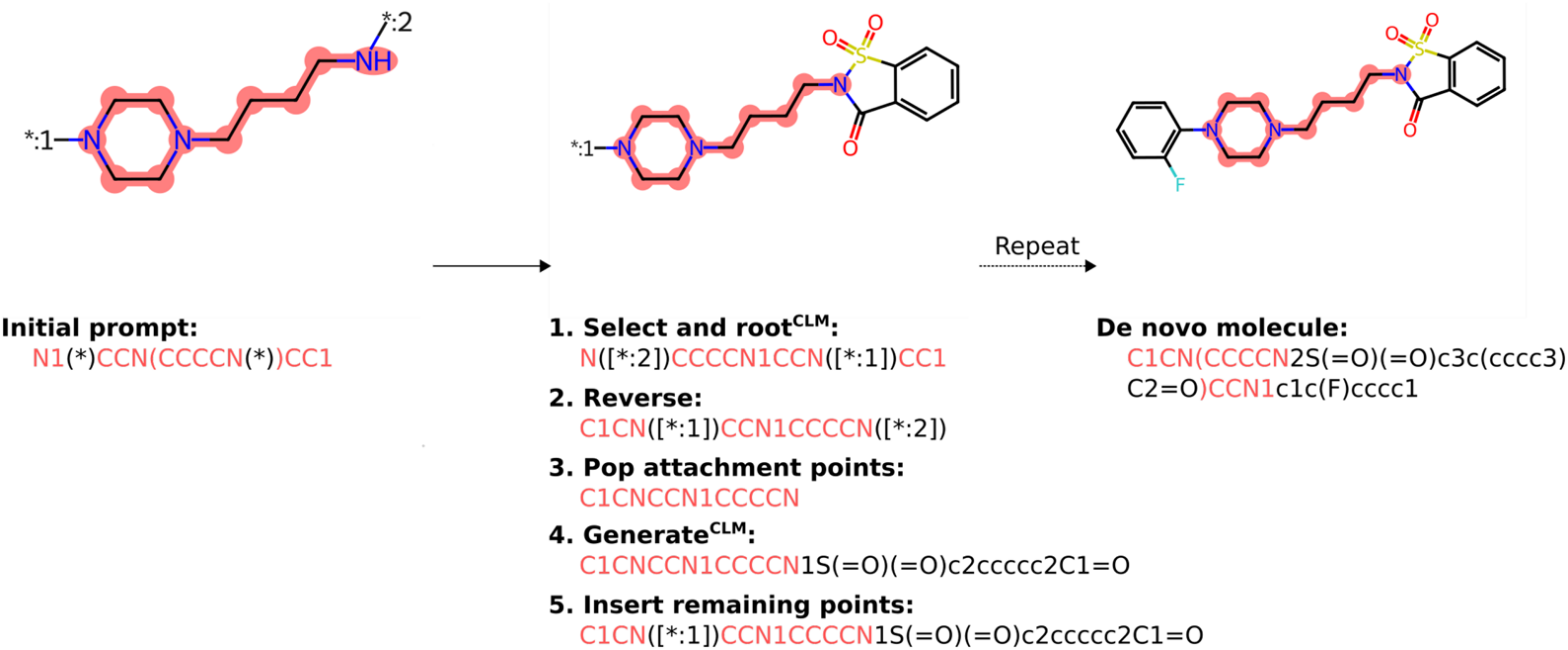
Schematic example of scaffold constrained decoration with PromptSMILES and a CLM. Attachment points are labelled with a number only for demonstrative purposes, in practice this is handled automatically by PromptSMILES. The CLM can optionally be used to optimize the SMILES re-arrangement in step 1 and is required for prompted de novo molecule generation in step 4

A scaffold *S* with multiple attachment points *A* can be represented in a SMILES string by adding branched dummy atoms (i.e., (*)), for example, a meta-substituted benzene can be represented as “c1c(*)cc(*)cc1”. The method of decoration is demonstrated in Fig. 1 and described in Algorithm 1. First, an attachment can be selected either in order of appearance in the string (canonical) or it can be randomly selected such that a batch of molecules contains a mixture of selected attachment points (shuffle). Then, the SMILES string can be generated to be rooted at this index using RDKit and then reversed such that it is now the last atom. Alternatively, several rooted randomized variations of SMILES representations can be generated [27], reversed, and the CLM likelihood of the full SMILES string calculated to select the variation most likely generated by the CLM (optimise). Note that because the SMILES string is only modified from the prompt onwards, it is trivial to remove and re-insert attachment points in the prompt for later iterations (as attachment points will not be modelled by a typical CLM). Then, the CLM samples new tokens at each timestep $x_{t}$ provided that a prompt token $s^{*}_{t}$ at that timestep *t* does not exist. Once SMILES generation has terminated due to the sampling of a stop token any remaining attachment points are inserted back into the string. This is process is then repeated until all attachment points have been sampled.

**Algorithm 1:**
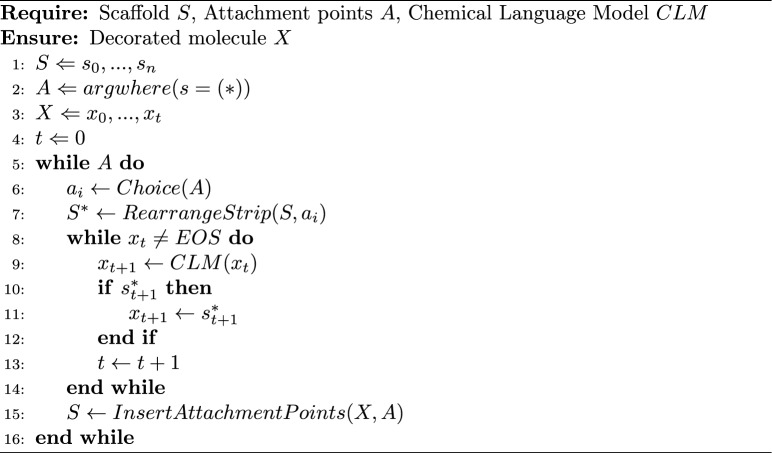
Decorating a scaffold using prompts

### Prompt-based fragment linking

Linking two fragments *F* together given a specified attachment point $A_{F}$ on each is a considerably easier problem, and is described in Algorithm 2. First, a fragment is chosen either based on the order of appearance (canonical) or randomly selected such that a batch contains a mixture of starting fragments (shuffle). Then, the first fragment SMILES undergoes the same procedure as a scaffold but given only one provided attachment point. The second fragment goes through the same procedure however, it is not reversed such that the attachment point is the first atom in the SMILES string. Following this, the CLM samples new tokens at each timestep $x_{t}$ provided there isn’t already a prompt token $f^{*}_{t}$ at that timestep *t*. Once SMILES generation has terminated due to the sampling of a stop token, the stop token is removed and the second fragment is simply concatenated to the generated SMILES string. Similar to scaffold decoration, the input fragments can be represented as a list of SMILES strings with one branched dummy atom each, for example, [“C1C(*)C1”, “n1(*)ccncc1”].

Linking more than two fragments *F* together given a single specified attachment point $A_{F}$ requires a slightly different process. For each fragment after the initial prompt fragment, the molecule is scanned and a fragment is inserted at each linker atom and the likelihood of the sequence being generated by the CLM is assessed. Such that the insertion point is selected to result in the highest overall sequence likelihood. Further fragments are inhibited from insertion at the indexes of a previous fragment. This approach also allows non-linear fragment linking i.e., A-X-C as well as A-X(-C)-B (see Figure B14 for demonstrative examples). The algorithm for this is shown in Algorithm 3. One caveat to this approach is that during RL the model may learn to generate the desired fragment before insertion, therefore, we implemented a simple check to skip fragment insertion if it already exists to help limit repeated fragments if they occur.

**Algorithm 2:**
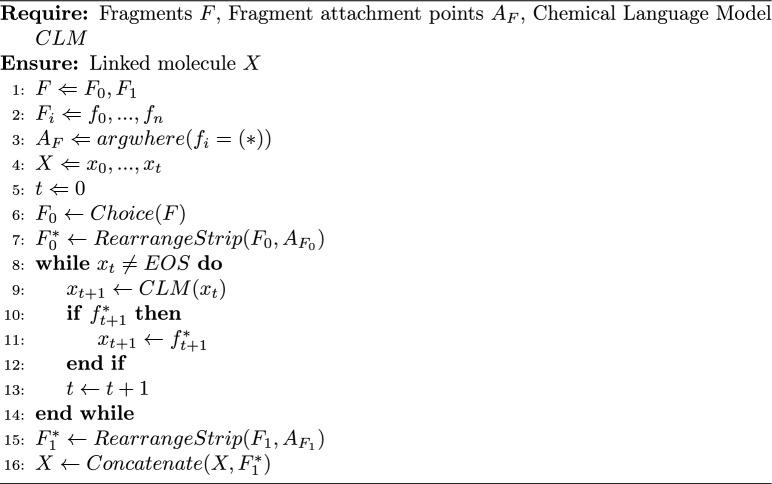
Linking two fragments together using prompts

**Algorithm 3:**
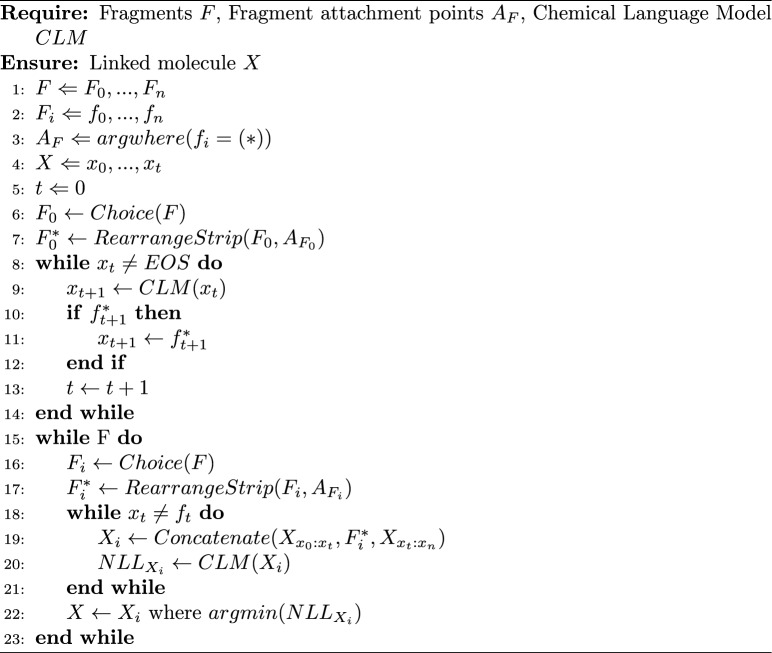
Linking more than two fragments together using prompts

### Reinforcement learning

The RL algorithm used in this work is the same as in REINVENT [6] for comparative reasons. Specifically, we set $\sigma =120$ as per the default in more recent versions of REINVENT [35, 36]. Note the implementation of this approach requires iterative sampling of the CLM and several forward passes of the network. Therefore, to ensure stable learning, RL is split into a collection phase without computing gradients, followed by an update phase given a completed SMILES string. Furthermore, we investigate two approaches to the frequency of network updates, one update per final SMILES string completion, or one for every iteration of prompt-based completion (multi). In the case of scaffold decoration, the completed SMILES string is used for the single update. For fragment linking, the initial fragment plus de novo linker SMILES is used for the single update, as using the completed SMILES would encourage the CLM to generate the fragments on its own accord. In all cases, the reward is calculated based on the completed SMILES.

### Implementation

**Fig. 2:**
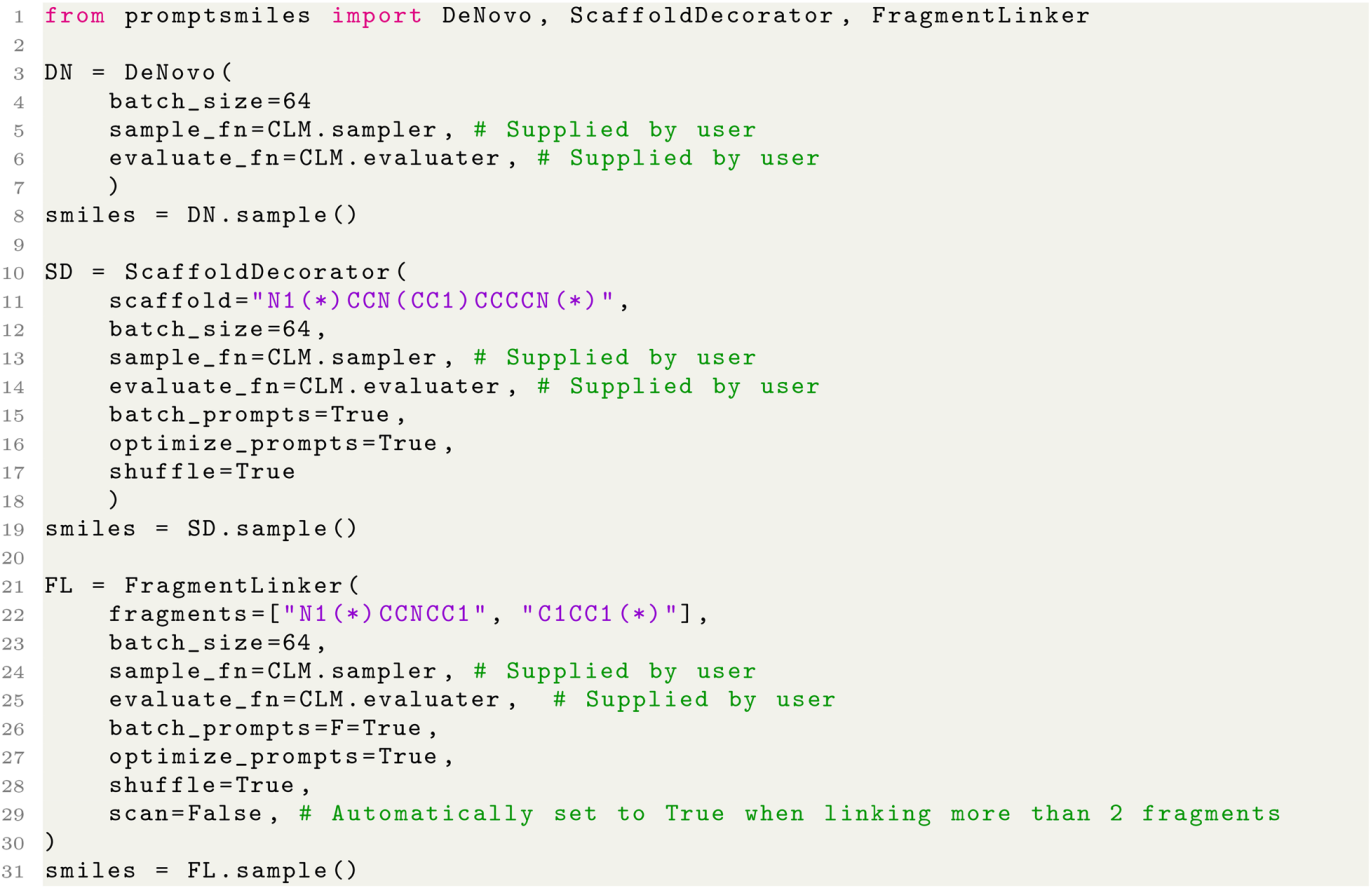
Code example of how to use PromptSMILES de novo generation (a wrapper of CLM sampling with no effect, for universal integration purposes), for scaffold decoration or for fragment linking

This approach is implemented with a python package named PromptSMILES. The software PromptSMILES simply automates the SMILES rearrangements described in this work given a function that conducts auto-regressive CLM SMILES generation from a prompt (or without) and a function that calculates the CLM likelihood of a SMILES string, facilitating easy integration with other libraries like the Transformers library in HuggingFace [37] or ACEGEN [38]. This was a design choice considering that different users may want to implement this approach with different CLM implementations, therefore it is packaged separately to any CLM. The experiments conducted here were generated using the SMILES-RNN repository with PromptSMILES integrated which also serves as an example for integration alongside the python code snippets in Fig. 2.

## Results

To investigate the performance of PromptSMILES for scaffold decoration and fragment linking, we compare performance on experiments conducted by SAMOA, MoLeR, LibINVENT, and LinkINVENT based on the reproducibility of their experiments and reported results. We also demonstrate the extended ability to conduct fragment linking between more than two fragments.

### Scaffold decoration of drug-like scaffolds at specified attachment points

**Fig. 3:**
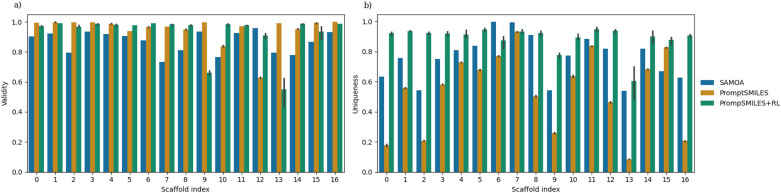
The (**a**) validity and (**b**) uniqueness of 10,000 de novo SMILES decorating 17 different SureChEMBL scaffolds by PromptSMILES in comparison to baseline SAMOA. PromptSMILES results in a higher ratio of valid SMILES but a lower number of unique molecules compared to SAMOA. This is rescued by using PromptSMILES+RL with a simple representative objective. PromptSMILES experiments were replicated 3 times

To investigate the baseline behaviour of our approach, we re-implemented the first experiment conducted by Langevin et al. [26] and compared the results to their method SAMOA. Whereby 17 drug discovery relevant scaffolds were extracted from SureChEMBL [39] with between 1 to 5 specified attachment points per scaffold used for scaffold-constrained molecule generation. We pre-trained an RNN with the same hyperparameters and training dataset as provided by the authors. Note that the training dataset does not contain any molecules with any of the 17 validation set scaffolds. Once trained, 10,000 scaffold-decorated molecules were sampled and the validity and uniqueness were measured as shown in Fig. 3 with examples shown in Figure B3.

For 16/17 scaffolds, PromptSMILES generates a higher fraction of valid de novo SMILES, however, for only 1 in 17 scaffolds does it generate a higher fraction of unique de novo SMILES. This is expected due to the limitation introduced by the iterative nature of prompt completion earlier described in the Methods: after decoration of the first attachment point the molecule is likely considered already complete for future iterations. This could potentially be improved by increasing sampling temperature, however, we proposed to see if the model was able to overcome this limitation by transfer learning to this new task via RL by using a simple representative objective task. The model was trained using the REINVENT algorithm to maximize the reward returned, whereby 1 was provided as the reward if a molecule was valid and unique, and 0 if the molecule was invalid or non-unique. The first 10,000 de novo SMILES generated are additionally plotted in Fig. 3 with examples shown in Figure B4. PromptSMILES+RL is able to increase the unique fraction of SMILES generated in all cases and beyond the baseline SAMOA method for 15/17 scaffolds while maintaining a high fraction of valid SMILES. Note that performance of different PromptSMILES parameters can be seen in Figure B1 and Figure B2. This indicates that purely prompt-based conditional generation can be used to decorate scaffolds of multiple attachment points (up to 5 in this experiment). From here on, PromptSMILES refers to PromptSMILES with RL for fine-tuning and conditional optimisation of a specified objective.

### Scaffold decoration to satisfy chemical reaction constraints

Beyond the ability to generate valid and unique de novo molecules adhering to a pre-defined scaffold, it is further of interest to be able to condition molecule generation according to some desirable endpoint, for example, predicted binding affinity or synthesizability. LibINVENT [28] is a encoder-decoder style architecture that translates an input scaffold into output decorations. Furthermore, the training dataset is curated by splitting molecules based on a set of reaction rules, therefore, encouraging the implicit learning of reaction rules and improving synthesizability of proposed de novo molecules. We re-implemented the first experiment conducted by the authors of LibINVENT. Firstly, we pre-train an RNN based on the same hyperparameters and training dataset provided by the authors. Note that we only take unique complete (non-sliced) molecules from the training dataset for training. Then we re-implement the RL objective tasks in MolScore [40] using the same trained QSAR model to predict the probability of Dopamine Receptor D2 (D_2_) activity and a re-implementation of the reaction filters. The RL algorithm used is the same as REINVENT and is likewise compared to LibINVENT over a run for 100 epochs.

In the first sub-experiment, PromptSMILES is trained to optimize scaffold decoration to increase the predicted probability of D_2_ activity (note exact reproduction was not possible due to the of lack diversity filter specification by the authors of LibINVENT, therefore, any difference in diversity filter used may also lead to slightly different results). Table 1 shows the results of this experiment compared to those reported for LibINVENT, with the top 3 molecules shown in Figure B10 and optimization curves shown in Figure B11. We further split the results based the parameters used for PromptSMILES i.e., if the prompt undergoes highest CLM likelihood optimisation (optimise) or not, if the attachment point is randomly selected (shuffle) or not, and whether network updates are per prompt completion (multi) or not. For the task of optimizing D_2_ predicted probability of activity without regard for synthesizability, PromptSMILES generates a higher number of successful compounds than LibINVENT in 7/8 configurations, resulting in a higher yield, and even with a higher average score in the resulting dataset in 2/8 configurations. Furthermore, if an example PromptSMILES configuration is run for more epochs to allocate a grace number of initial RL epochs to the transfer learning task of iterative prompting, Table 1 shows that average score increases further.

**Table 1:** PromptSMILES optimization for a QSAR model with no reaction filters

Method	No. successful compounds	Yield	Average score
LibINVENT	10,510 ± 69	0.821 ± 0.005	0.722 ± 0.005
PromptSMILES	10,748 ± 215	0.831 ± 0.016	0.636 ± 0.044
PromptSMILES (optimise)	**11,075 ± 88**	**0.857 ± 0.007**	0.657 ± 0.006
PromptSMILES (multi)*	10,622 ± 310	0.822 ± 0.024	0.714 ± 0.035
PromptSMILES (optimise,multi)	10,833 ± 183	0.839 ± 0.014	0.727 ± 0.007
PromptSMILES (shuffle)	10,130 ± 460	0.784 ± 0.036	0.629 ± 0.055
PromptSMILES (optimise,shuffle)	10,781 ± 206	0.834 ± 0.016	0.686 ± 0.011
PromptSMILES (multi,shuffle)	10,636 ± 162	0.823 ± 0.013	**0.732 ± 0.003**
PromptSMILES (optimise,multi,shuffle)	10,704 ± 113	0.828 ± 0.008	0.718 ± 0.011
PromptSMILES* (Epoch: 100–200)	*10,342 ± 90*	*0.816 ± 0.007*	**0.762 ± 0.009**

Best value is highlighted in bold if better than the baseline approach, and italics otherwise. This is repeated for Epochs 100-200

PromptSMILES experiments were replicated 3 times

*Longer run from selected configuration shows continued improvement in average score

In the second sub-experiment, PromptSMILES is trained to both optimize scaffold decoration to increase the predicted probability of D_2_ activity, as well as, adhere to a selective reaction filter. The selective reaction filter specifies that the first attachment point should be decorated via an amide coupling and the second via a Buchwald-Hartwig reaction. Otherwise the same parameters apply as in the first part of the experiment. Table 2 shows that for 6/8 configurations, more successful compounds are found than LibINVENT resulting in a higher yield, and with a higher average score (examples shown in Figure B12 and optimisation curves in Figure B13). Meanwhile, the ratio of satisfied reaction filters doesn’t quite outperform LibINVENT for any configuration. However, considering that in comparison there is no pre-training based on molecules already sliced by these reaction rules as in LibINVENT, PromptSMILES manages to first adapt to the task of scaffold decoration then learn to decorate the scaffold such that in the best case almost 80% of molecules satisfy both reaction filters. Similar to the first sub-experiment, this improves for a selected configuration to approximately 83%, if the first epochs are allocated to transfer learning.

**Table 2:** PromptSMILES optimization for a QSAR model with a selective reaction filter

Method	No. successful compounds	Yield	Average score	Ratio of satisfied reaction filters
LibINVENT	10,454 ± 192	0.817 ± 0.015	0.729 ± 0.008	**0.892 ± 0.032**
PromptSMILES	10,739 ± 320	0.831 ± 0.025	0.695 ± 0.012	0.730 ± 0.027
PromptSMILES (optimise)	10,410 ± 193	0.805 ± 0.015	0.682 ± 0.003	0.693 ± 0.021
PromptSMILES (multi)	10,569 ± 175	0.818 ± 0.014	0.748 ± 0.020	0.775 ± 0.016
PromptSMILES (optimise,multi)	10,631 ± 179	0.822 ± 0.014	0.756 ± 0.010	0.778 ± 0.012
PromptSMILES (shuffle)	10,444 ± 359	0.808 ± 0.028	0.708 ± 0.023	0.715 ± 0.038
PromptSMILES (optimise,shuffle)	**11,162 ± 108**	**0.864 ± 0.008**	0.697 ± 0.005	0.766 ± 0.019
PromptSMILES (multi,shuffle)*	10,816 ± 157	0.837 ± 0.012	**0.775 ± 0.010**	*0.792 ± 0.010*
PromptSMILES (optimise,multi,shuffle)	10,733 ± 192	0.830 ± 0.015	0.748 ± 0.016	0.788 ± 0.014
PromptSMILES* (Epoch: 100–200)	**10,736 ± 40**	**0.830 ± 0.003**	**0.818 ± 0.005**	*0.827 ± 0.004*

Best value is highlighted in bold if better than the baseline approach, and italics otherwise. This is repeated for Epochs 100-200

PromptSMILES experiments were replicated 3 times

*Longer run from selected configuration shows continued improvement in average score and satisfaction of reaction filters

### Scaffold decoration and objective optimisation with unspecified attachment points

In some cases, there may not be any specified or desired scaffold attachment points. Our approach can be trivially extended to this scenario by simply viewing every atom with available valence as being an attachment point. To demonstrate this, we re-implement the scaffold-constrained GuacaMol benchmarks proposed by Maziarz et al. [31]. This benchmark specifies four scaffolds for scaffold-constrained generation and respective scoring functions to evaluate de novo molecules. The prior used was trained on the GuacaMol dataset and the optimization algorithm used is the same as in REINVENT, run for 500 epochs. For each scaffold, we specify every atom as an available attachment point (see Figure B5). The results of this benchmark are shown in Table 3 in comparison to other scaffold-constrained generative methods as reported. Here, as there are more attachment points and no diversity filters in the scoring functions, we run PromptSMILES with shuffle and prompt optimisation and only investigate the use of multiple RL updates or not. Although PromptSMILES gets the lowest overall score on the benchmark, it still scores highly with a comparable score of 0.87 or 0.90 and a comparable quality metric of 0.53 for the top 100 proposed compounds. However, there are some nuances to the use of SMILES prompts for these benchmark tasks. For example, for the factor Xa task, the similarity objective should require the model to connect two atoms of the substructure to form an oxazolidinone. However, this requires that the CLM appends a ring connection to one attachment point without closing it, followed by a closing ring connection to another attachment point at a later iteration. This process would result in an invalid SMILES string in-between iterations. This is a limitation of our current implementation which requires RDKit to parse a molecule at each iteration, rather than the concept of this approach. For, factor Xa, this can be circumvented by shortening the scaffold-prompt so that the CLM extends a single attachment point and can open and close a ring within one iteration (see Figure B5). Table 3 shows that by simplifying the factor Xa scaffold, the quality metric matches (and sometimes exceeds) state-of-the-art compared to other approaches albeit a limited comparison. The same nuance applies to macrocyclisation of lorlati, however, this can not be circumvented as easily and requires an implementation without requiring valid molecules at each iteration. This task is predominantly responsible for decreasing the average score (see Figure B6 and Figure B7). Interestingly, the use of multiple RL updates and therefore stronger optimisation ability increased the score but decreased the chemical quality of the top 100 compounds. This is suggestive of mode collapse which may require a diversity filter to circumvent. We also note that most objectives are optimized by approximately 300 epochs (see Figure B6) which required $237\pm 180$ minutes ($215\pm 169$ for multi) on a single NVIDIA RTX 4090 with the scoring functions utilising 1 CPU core. This is significantly more efficient than the reported value of MoLeR of 6 to 130 GPU hours [31].

**Table 3:** Results on four additional scaffold-based GuacaMol tasks

Method	Score	Quality
CDDD+MSO	0.92	0.59
MNCE-RL	0.95	0.47
MoLeR+MSO	0.93	0.63
PromptSMILES (optimise,shuffle)	0.87 ± 0.10 (0.88 ± 0.10)	0.53 ± 0.26 (0.61 ± 0.19)
PromptSMILES (optimise,shuffle,multi)	0.90 ± 0.09 (0.90 ± 0.09)	0.46 ± 0.33 (0.45 ± 0.33)

PromptSMILES experiments were replicated 3 times

Numbers reported in parenthesis are with the simplified factor Xa scaffold

### Linking two fragments

In order to assess the performance of linking two fragments together, we re-implement the same experiment as for LinkINVENT [29]. LinkINVENT is the same encoder-decoder RNN as used in LibINVENT, however, the model is trained to translate two fragments into a linker. Similarly, we pre-train a decoder-only RNN using the same model hyperparameters and training dataset but extracting unique complete (non-sliced) molecules. This experiment is divided into three sub-experiments: (1) to control linker length, (2) to control linker linearity and (3) to control linker flexability. All RL objectives were re-implemented and available to use with MolScore [40].

**Fig. 4:**
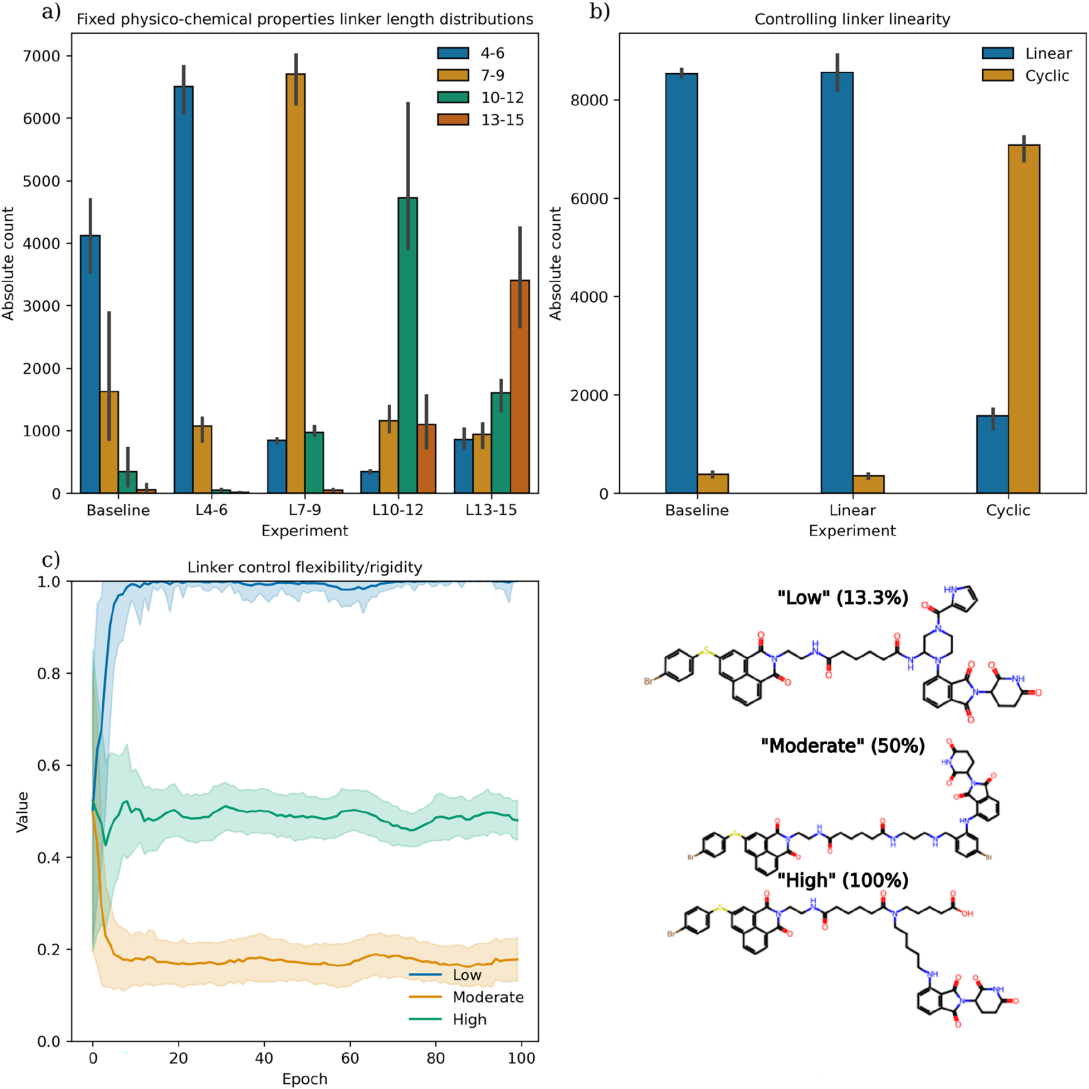
Fragment linking using PromptSMILES (optimise,shuffle) while controlling linker properties via RL as conducted in LinkINVENT. The objectives can be categorised into three experiments (**a**) controlling the linker length, (**b**) controlling the linker linearity, and (**c**) controlling the linker flexibility i.e., ratio of rotatable bonds where low is 0–30%, moderate is 40–60% and high is 70–100%. Random samples taken from each objective in experiment (c) are shown. PromptSMILES experiments were replicated 3 times

Figure 4 shows the results of PromptSMILES (optimise,shuffle) for each sub-experiment. This shows that the results are comparable to those reported by LinkINVENT i.e., RL is able to teach the decoder-only model to generate linkers that satisfy a number of different properties. Therefore, PromptSMILES additionally enables the use of a pre-trained CLM for linker generation.

### Linking more than two fragments

The ability to link more than two fragments together extends the practical utility to scenarios where maintaining more sub-structures is desirable. For example, if a molecule extends into more than two important sub-pockets in a binding pocket that should be maintained, but a new scaffold is of interest. PromptSMILES also extends to this scenario with some adaptation. To test the generalization of PromptSMILES to link more than two fragments, we run an experiment to attempt to recover Atorvastatin, a HMG-CoA reductase inhibitor prescribed for the treatment of hypercholesterolemia [41, 42]. The RL objective was the Tanimoto similarity to the reference compound.

**Fig. 5:**
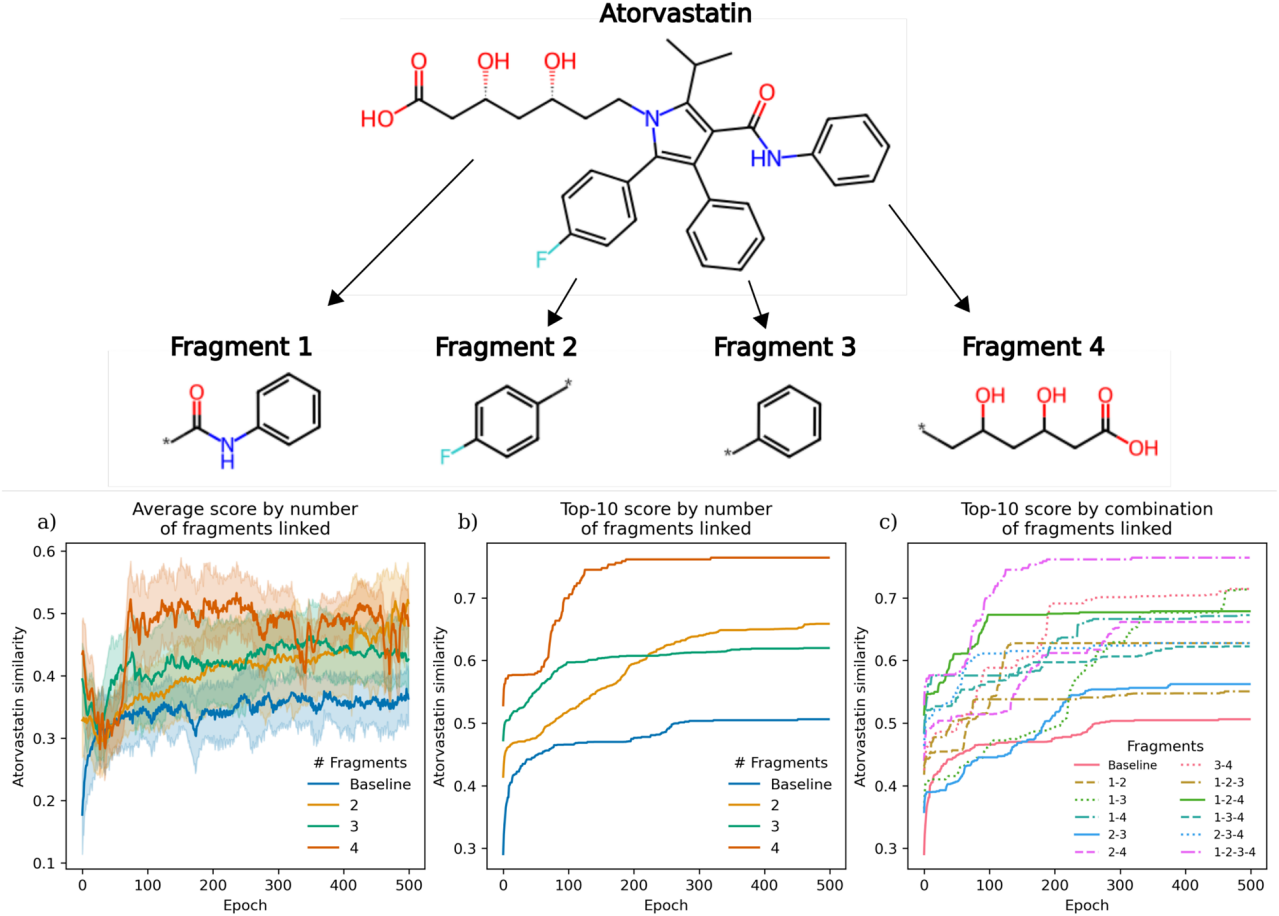
Fragment linking more than two fragments using PromptSMILES (optimise,shuffle) while maximizing similarity to Atorvastatin via RL. All combinations of the four fragments shown are investigated. **a** Average similarity to Atorvastatin by the number of fragments linked, **b** Top-10 similarity, and **c** the Top-10 similarity by the precise combination of fragments linked

Figure 5 shows the score achieved when running baseline optimization (de novo generation) or constrained to linking two, three or even four sub-structures of the reference compounds as fragments. Firstly, constraining generation to a larger number of predefined fragments increases basal similarity to Atorvastatin at Epoch 0 as expected, depicting the benefit of specifying apriori knowledge of chemistry if known or desired. Secondly, regardless of the number of fragments, RL is still able to learn how to improve the reward and increase similarity to Atorvastatin. Interestingly, Fig. 5a and b shows that the use of two fragments eventually achieves a higher score than the use of three fragments. This also corresponds to a switch in approach from Algorithm 2 to 3 which is conceptually more difficult due to a reliance on the stochasticity of the CLM with respect to the placement of fragments to the linker. Additionally, Figure B14 shows that the CLM still learns to generate the unspecified fragments. But, specifying more apriori knowledge with four fragments still achieves a higher score overall and could prove a useful capability in practice.

Also observable is with an increasing number of fragments there is an increasing delay in learning while the model adapts to the new type of task. As shown in Fig. 5a, the baseline approach starts increasing similarity immediately, however, on average linking two, three, or four fragments first sees a dip in Atorvastatin similarity at approximately 30, 40 and 50 Epochs before an increase, respectively. In the context of using specially curated grammars, datasets or architectures, we think that this initial delay in learning is a worthwhile trade-off and still more efficient overall.

### Switching between scaffold decoration and fragment linking

**Fig. 6:**
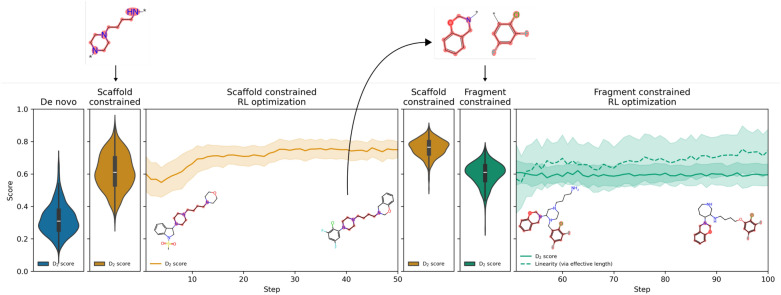
Flexability of PromptSMILES (optimise, shuffle) on the LibINVENT D_2_ experiment. From left to right: Distribution of D_2_ activity score of de novo molecules sampled from the unconditioned prior, distribution of D_2_ activity score of scaffold constrained molecules sampled from the unconditioned prior, RL optimization of LibINVENT D_2_ task with scaffold constraints, distribution of D_2_ activity score of scaffold constrained molecules sampled from the trained agent at 50 steps, distribution of D_2_ activity score of fragment constrained molecules sampled from the trained agent at 50 steps (fragments selected from a promising generated molecule), RL optimization of LibINVENT D_2_ task with an additional linker linearity objective and fragment constraints

One key advantage of PromptSMILES is the ability to switch from scaffold decoration to fragment linking and vice versa without the need to use an alternative model or re-train a model. We have demonstrated this by repeating the LibINVENT experiment of optimizing the D_2_ receptor activity score but switching from de novo sampling, to scaffold decoration, to fragment linking, as well as introducing a new objective to the task part way, much like curriculum learning. Figure 6 shows the evolution of this practical flexibility experiment. This shows that simply adding the D_2_-based scaffold constraints increases the distribution of D_2_ activity score for 1000 molecules sampled from the unconditioned prior. Then, RL is used to further optimize this score for 50 steps and a further 1000 molecules are sampled with scaffold constraints demonstrating further improvement of the score. A generated molecule at step 50 is chosen to mimic a compound that has promising decorative groups and is now of interest to search for new scaffolds/linkers. The fragments extracted are used as the basis for fragment linking, the same agent is sampled with 1000 fragment linked molecules showing a higher D_2_ score distribution than de novo molecules sampled from the unconditioned prior. Similar to LinkINVENT, a linker linearity objective is introduced to ensure a certain degree of linearity. RL is used to further optimize both these objectives showing maintenance of D_2_ score and an increase in linker linearity. Overall, this demonstrates the flexibility to switch between de novo design, scaffold decoration, and fragment linking with the same chemical agent.

## Conclusion

In this work, we have proposed an iterative method of prompting we call PromptSMILES to achieve scaffold decoration and fragment linking (a.k.a. scaffold hopping) with CLMs in drug design. In combination with RL, we show that decoder-only CLMs can quickly adapt to the task of iterative prompting as well as maintain the ability to optimize arbitrary objectives. This was done by demonstrating comparable or improved performance for a variety of orthogonal approaches and different objectives. The ability to link more than two fragments in a non-linear way is a further extension to practical utility. Crucially, this method does not require a bespoke architecture, bespoke grammar, or even retraining of a SMILES-based decoder-only model. Therefore, with the same pre-trained CLM, you can conduct de novo generation, scaffold decoration, and fragment linking.

### Supplementary Information

Supplementary Material 1.

## Data Availability

The code used to manipulate SMILES prompts used in this work has been packaged and deposited on PyPI for convenience (https://pypi.org/project/promptsmiles/), or can be found on GitHub (https://github.com/compsciencelab/PromptSMILES). The code used for integration with a chemical language model and reinforcement learning can be found on GitHub (https://github.com/MorganCThomas/SMILES-RNN).

## Appendix A

### Experiments

We endeavoured to follow model architectures and parameters to compared models in each experiment where possible, with the exception that we always trained the respective RNN with SMILES augmentation by 10-fold restricted randomization [43]. This was done due to the fact that PromptSMILES rearranges SMILES strings forming non-canonical representations. All objectives were implemented with MolScore [40], details can be found in the configuration files (https://github.com/MorganCThomas/MolScore/tree/main/molscore/configs/PromptSMILES).

### Scaffold decoration of drug-like scaffolds at specified attachment points

This experiment was a re-implementation based on an experiment by Langevin et al. [26] to compare to the reported results for SAMOA made available by the authors.

**CLM** The CLM used in this experiment was an RNN with 3 layers of GRU cells, a hidden dimension size of 512, embedding size of 128 and Adam optimizer.

**Dataset and Training** The CLM was trained on the ChEMBL without SureChEMBL scaffolds provided by the authors, with a batch size of 128 and learning rate of 0.001 for 5 epochs.

### Scaffold decoration to satisfy chemical reaction constraints

This experiment was a re-implementation based on an experiment by Fialková et al. [28] to compare to the reported results for LibINVENT.

**CLM** The CLM used in this experiment was an RNN with 3 layers of LSTM cells, a hidden dimension size of 512, embedding size of 256 and Adam optimizer with a dropout rate of 0.1.

**Dataset and Training** The CLM was trained on the ChEMBL dataset provided by the authors. As this dataset comprised of tuples of scaffold, decorations, and complete molecules, we only take the unique set of complete molecules for training. The model was trained with a batch size of 128 and learning rate of 0.001 for 5 epochs.

### Scaffold decoration and objective optimisation with unspecified attachment points

This experiment was a re-implementation based on the benchmark proposed by Maziarz et al. [31] to compare to the reported results for MoLeR. As the architecture differs between CLMs and the graph-based neural network used in MoLeR, we used this as an example of using an already trained CLM from previous work [13].

**CLM** The CLM used in this experiment was an RNN with 3 layers of LSTM cells, a hidden dimension size of 512, embedding size of 512 and Adam optimizer with a dropout rate of 0.2.

**Dataset and Training** The CLM was trained on the GuacaMol train dataset [15] with a batch size of 512 and learning rate of 0.001 for 10 epochs.

### Linking two fragments

This experiment was a re-implementation based on an experiment by Guo et al. [29] to compare to the reported results for LinkINVENT.

**CLM** The CLM used in this experiment was an RNN with 3 layers of LSTM cells, a hidden dimension size of 512, embedding size of 256 and Adam optimizer.

**Dataset and Training** The CLM was trained on the ChEMBL dataset provided by the authors. As this dataset comprised of tuples of fragments, linker, and complete molecules, we only take the unique set of complete molecules for training. The model was trained with a batch size of 128 and learning rate of 0.001 for 5 epochs.

### Linking more than two fragments

This new experiment was to demonstrate the ability to link more than two fragments together. The CLM, dataset and training was the same as for the previous experiment linking two fragments together.

### Switching between scaffold decoration and fragment linking

This new experiment demonstrated the flexibility to switch between de novo and constrained generation with the same CLM. This CLM, dataset and training was the same as in the comparison to LibINVENT. The first objective used for RL is the same as the first sub-experiment in the comparison to LibINVENT (i.e., without the use of reaction filters). For the second objective, a linker linearity scoring parameter is added resulting in a score of 1 if the linker length ratio is above 0.5, and 0 otherwise. This score is then combined with the others via the product aggregation.

## References

[1] Ress DC, Congreve M, Murray CW, Carr R (2004). Fragment-based lead discovery. Nat Rev Drug Discovery.

[2] Sun X, Gao H, Yang Y, He M, Wu Y, Song Y, Tong Y, Rao Y (2019). Protacs: great opportunities for academia and industry. Signal Transduct Target Ther.

[3] Hu Y, Stumpfe D, Bajorath J (2017). Recent advances in scaffold hopping. J Med Chem.

[4] Segler MHS, Kogej T, Tyrchan C, Waller MP (2018). Generating focused molecule libraries for drug discovery with recurrent neural networks. ACS Cent Sci.

[5] Amabilino S, Pogány P, Pickett SD, Green DVS (2020). Guidelines for recurrent neural network transfer learning-based molecular generation of focused libraries. J Chem Inf Model.

[6] Olivecrona M, Blaschke T, Engkvist O, Chen H (2017). Molecular de-novo design through deep reinforcement learning. J Cheminformatics.

[7] Popova M, Isayev O, Tropsha A (2018). Deep reinforcement learning for de novo drug design. Sci Adv.

[8] Blaschke T, Engkvist O, Bajorath J, Chen H (2020). Memory-assisted reinforcement learning for diverse molecular de novo design. J Cheminformatics.

[9] Korshunova M, Huang N, Capuzzi S, Radchenko DS, Savych O, Moroz YS, Wells CI, Willson TM, Tropsha A, Isayev O (2022). Generative and reinforcement learning approaches for the automated de novo design of bioactive compounds. Commun Chem.

[10] Bjerrum EJ, Margreitter C, Blaschke T, Castro RL-R (2022). Faster and more diverse de novo molecular optimization with double-loop reinforcement learning using augmented smiles. J Comput-Aided Mol Design.

[11] Guo J, Schwaller P (2023) Augmented memory: capitalizing on experience replay to accelerate de novo molecular design. arXiv

[12] Svensson HG, Tyrchan C, Engkvist O, Chehreghani MH (2023) Utilizing reinforcement learning for de novo drug design. arXiv

[13] Thomas M, O’Boyle NM, Bender A, Graaf C (2022). Augmented hill-climb increases reinforcement learning efficiency for language-based de novo molecule generation. J Cheminformatics.

[14] Atance SR, Diez JV, Engkvist O, Olsson S, Mercado R (2022). De novo drug design using reinforcement learning with graph-based deep generative models. J Chem Inf Model.

[15] Brown N, Fiscato M, Segler MHS, Vaucher AC (2019). Guacamol: benchmarking models for de novo molecular design. J Chem Inf Model.

[16] Polykovskiy D, Zhebrak A, Sanchez-Lengeling B, Golovanov S, Tatanov O, Belyaev S, Kurbanov R, Artamonov A, Aladinskiy V, Veselov M, Kadurin A, Johansson S, Chen H, Nikolenko S, Aspuru-Guzik A, Zhavoronkov A (2020). Molecular sets (moses): a benchmarking platform for molecular generation models. Front Pharmacol.

[17] Huang K, Fu T, Gao W, Zhao Y, Roohani Y, Leskovec J, Coley CW, Xiao C, Sun J, Zitnik M (2021) Therapeutics data commons: Machine learning datasets and tasks for drug discovery and development. 10.48550/arXiv.2102.09548. http://arxiv.org/abs/2102.09548

[18] Gao W, Fu T, Sun J, Coley C.W (2022) Sample efficiency matters: a benchmark for practical molecular optimization. arXiv 10.48550/arxiv.2206.12411

[19] Martinelli DD (2022). Generative machine learning for de novo drug discovery: a systematic review. Comput Biol Med.

[20] Merk D, Grisoni F, Friedrich L, Schneider G (2018). Tuning artificial intelligence on the de novo design of natural-product-inspired retinoid x receptor modulators. Commun Chem.

[21] Li X, Xu Y, Yao H, Lin K (2020). Chemical space exploration based on recurrent neural networks: applications in discovering kinase inhibitors. J Cheminformatics.

[22] Yang Y, Zheng S, Su S, Zhao C, Xu J, Chen H (2020). Syntalinker: automatic fragment linking with deep conditional transformer neural networks. Chem Sci.

[23] Grisoni F, Huisman BJH, Button AL, Moret M, Atz K, Merk D, Schneider G (2021). Combining generative artificial intelligence and on-chip synthesis for de novo drug design. Sci Adv.

[24] Hua Y, Fang X, Xing G, Xu Y, Liang L, Deng C, Dai X, Liu H, Lu T, Zhang Y, Chen Y (2022). Effective reaction-based de novo strategy for kinase targets: a case study on mertk inhibitors. J Chem Inf Model.

[25] Moret M, Angona IP, Cotos L, Yan S, Atz K, Brunner C, Baumgartner M, Grisoni F, Schneider G (2023). Leveraging molecular structure and bioactivity with chemical language models for de novo drug design. Nat Commun.

[26] Langevin M, Minoux H, Levesque M, Bianciotto M (2020). Scaffold-constrained molecular generation. J Chem Inf Model.

[27] Arús-Pous J, Patronov A, Bjerrum EJ, Tyrchan C, Reymond JL, Chen H, Engkvist O (2020). Smiles-based deep generative scaffold decorator for de-novo drug design. J Cheminformatics.

[28] Fialková V, Zhao J, Papadopoulos K, Engkvist O, Bjerrum EJ, Kogej T, Patronov A (2022). Libinvent: reaction-based generative scaffold decoration for in silico library design. J Chem Inf Model.

[29] Guo J, Knuth F, Margreitter C, Janet JP, Papadopoulos K, Engkvist O, Patronov A (2023). Link-invent: generative linker design with reinforcement learning. Digital Discovery.

[30] Yang Y, Zhang R, Li Z, Mei L, Wan S, Ding H, Chen Z, Xing J, Feng H, Han J, Jiang H, Zheng M, Luo C, Zhou B (2020). Discovery of highly potent, selective, and orally efficacious p300/cbp histone acetyltransferases inhibitors. J Med Chem.

[31] Maziarz K, Jackson-Flux H, Cameron P, Sirockin F, Schneider N, Stiefl N, Segler M, Brockschmidt M (2021) Learning to extend molecular scaffolds with structural motifs

[32] Noutahi E, Gabellini C, Craig M, Lim JSC, Tossou P (2023) Gotta be safe: a new framework for molecular design. arXiv

[33] OpenAI: (2023) Gpt-4 technical report. arXiv 10.48550/arXiv.2303.08774

[34] Weininger D (1988). Smiles, a chemical language and information system: 1: introduction to methodology and encoding rules. J Chem Inf Comput Sci.

[35] Blaschke T, Arús-Pous J, Chen H, Margreitter C, Tyrchan C, Engkvist O, Papadopoulos K, Patronov A (2020). Reinvent 2.0: an ai tool for de novo drug design. J Chem Inf Model.

[36] Loeffler HH, He J, Tibo A, Janet JP, Voronov A, Mervin L, Engkvist O (2023) Reinvent4: modern ai-driven generative molecule design. chemRxiv 10.26434/CHEMRXIV-2023-XT65X 10.1186/s13321-024-00812-5 PMC10882833 38383444

[37] Wolf T, Debut L, Sanh V, Chaumond J, Delangue C, Moi A, Cistac P, Ma C, Jernite Y, Plu J, Xu C, Le Scao T, Gugger S, Drame M, Lhoest Q, Rush AM (2020) Transformers: state-of-the-art natural language processing, pp. 38–45. Association for Computational Linguistics. https://www.aclweb.org/anthology/2020.emnlp-demos.6

[38] Bou A, Thomas M, Dittert S, Ramírez CN, Majewski M, Wang Y, Patel S, Tresadern G, Ahmad M, Moens V, et al Acegen: a torchrl-based toolkit for reinforcement learning in generative chemistry. In: ICLR 2024 Workshop on Generative and Experimental Perspectives for Biomolecular Design

[39] Papadatos G, Davies M, Dedman N, Chambers J, Gaulton A, Siddle J, Koks R, Irvine SA, Pettersson J, Goncharoff N, Hersey A, Overington JP (2016). Surechembl: a large-scale, chemically annotated patent document database. Nucleic Acids Res.

[40] Thomas M, O’Boyle NM, Bender A, Graaf C (2023) Molscore: a scoring and evaluation framework for de novo drug design. chemRxiv 10.26434/CHEMRXIV-2023-C4867 10.1186/s13321-024-00861-w PMC11141043 38816825

[41] Roth BD (2002). The discovery and development of atorvastatin, a potent novel hypolipidemic agent. Prog Med Chem.

[42] Istvan ES, Deisenhofer J (2001). Structural mechanism for statin inhibition of hmg-coa reductase. Science.

[43] Arús-Pous J, Johansson SV, Prykhodko O, Bjerrum EJ, Tyrchan C, Reymond J-L, Chen H, Engkvist O (2019). Randomized smiles strings improve the quality of molecular generative models. J Cheminformatics.

