# Supplementary material for: PromptSMILES: prompting for scaffold decoration and fragment linking in chemical language models
Source: J Cheminform. 2024 Jul 4;16:77. doi: 10.1186/s13321-024-00866-5 (PMC11225391; doi:10.1186/s13321-024-00866-5)
Supplement: Supplementary file 1 — Supplementary Material 1. [file 13321_2024_866_MOESM1_ESM.pdf]

Appendix B Supplementary figures

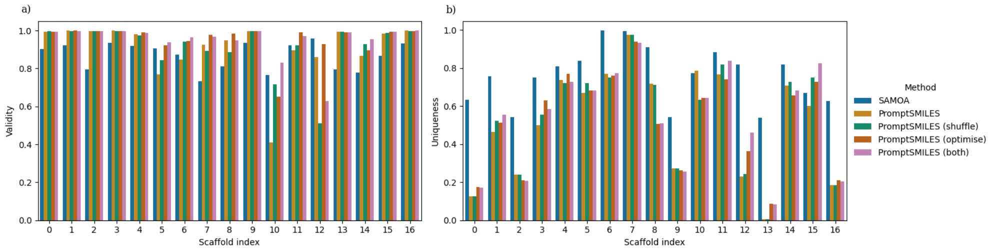

Fig. B1 The (a) validity and (b) uniqueness of 10,000 *de novo* SMILES decorating 17 different SureChEMBL scaffolds by PromptSMILES in comparison to baseline SAMOA. Different parameters for PromptSMILES are explored.

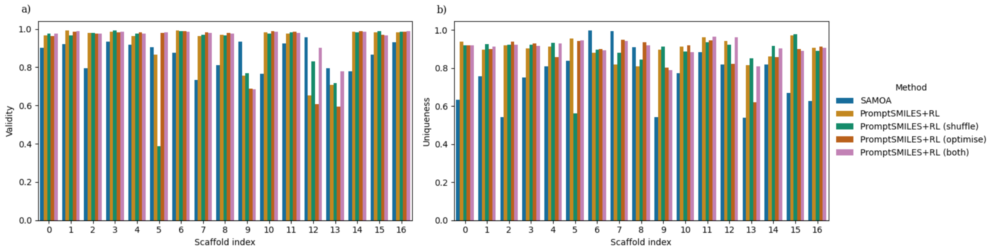

Fig. B2 The (a) validity and (b) uniqueness of 10,000 *de novo* SMILES decorating 17 different SureChEMBL scaffolds by PromptSMILES+RL in comparison to baseline SAMOA. Different parameters for PromptSMILES+RL are explored.

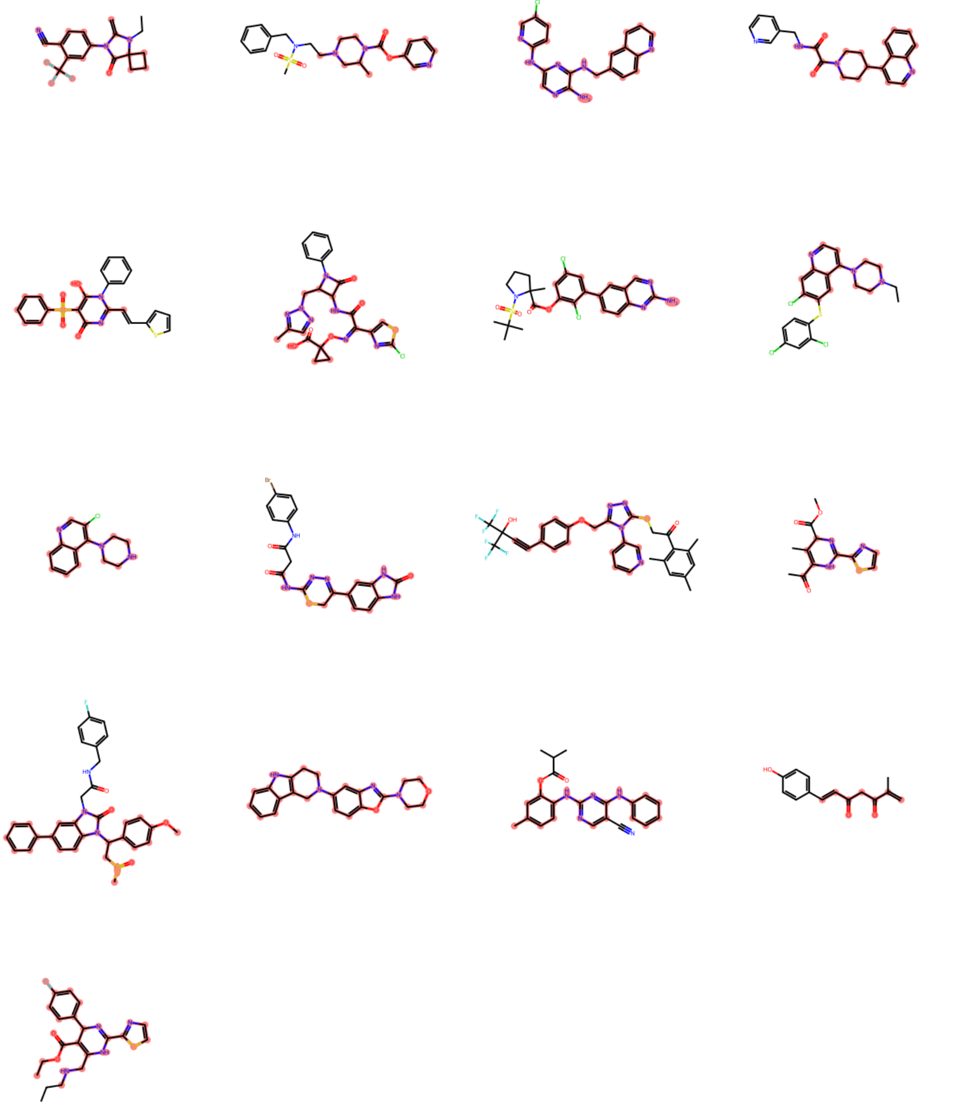

Fig. B3 Random example of a PromptSMILES decorated scaffold for each of the 17 reference scaffolds.

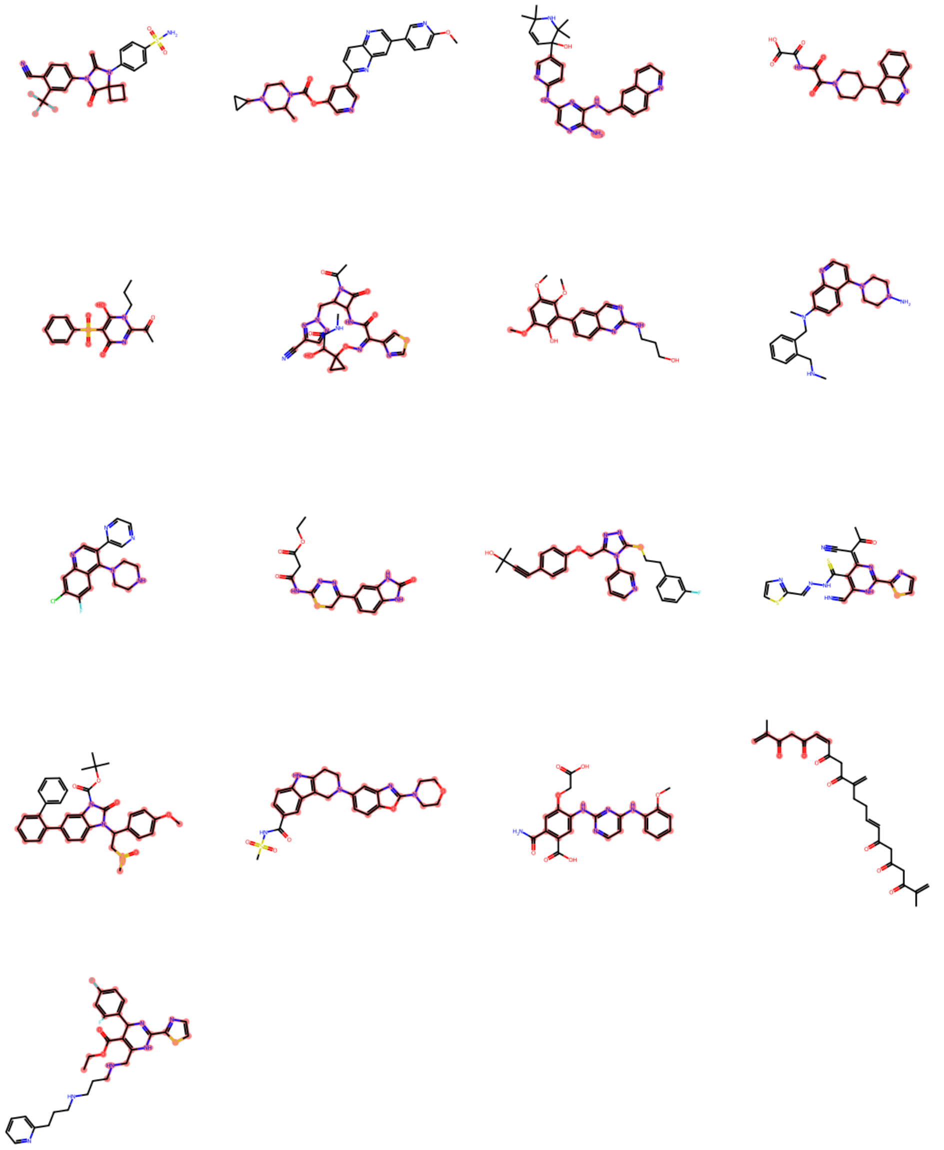

Fig. B4 Random example of a PromptSMILES+RL decorated scaffold for each of the 17 reference scaffolds. Note the RL objective is simply for a molecule to be valid and unique and therefore leads to undirected exploration.

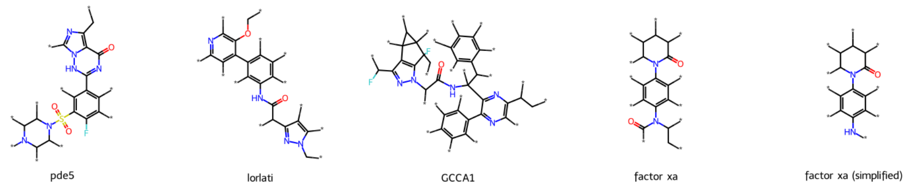

Fig. B5 Scaffolds and respective attachment point used to seed scaffold generation of PromptSMILES for the proposed GuacaMol Scaffolds benchmark. Note that an additional scaffold was used for factor Xa by pruning the scaffold to allow SMILES to open a ring in the desired location based on the objective.

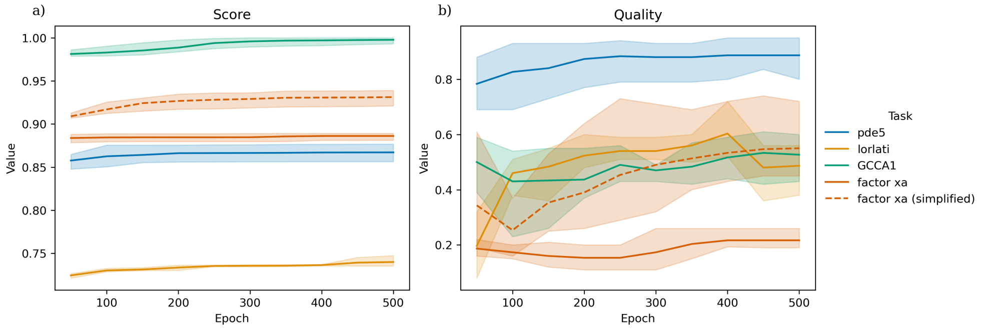

Fig. B6 Performance on the GuacaMol Scaffolds benchmark by epoch for PromptSMILES with optimise and shuffle. In some cases the score is already optimized after approximately 200 epochs, however, in further epochs are required to identify quality molecules (e.g., lorlati). Note that by simplifying the factor Xa scaffold, both a higher score and higher quality of *de novo* compounds is achieved.

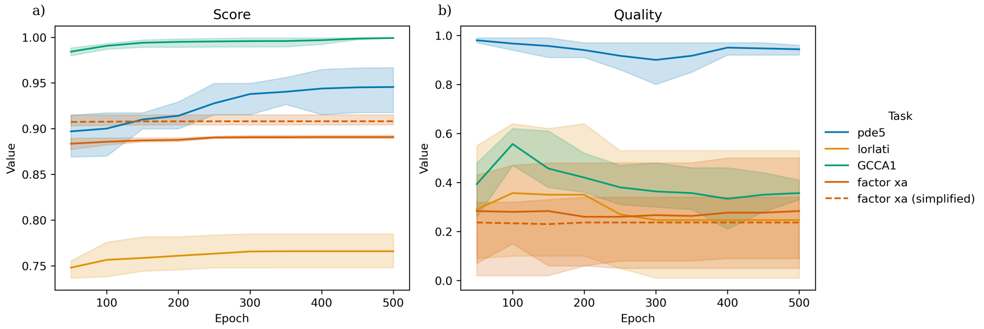

Fig. B7 Performance on the GuacaMol Scaffolds benchmark by epoch or PromptSMILES with optimise, shuffle and multi. In some cases the score is already optimized after approximately 200 epochs, however, in further epochs are required to identify quality molecules (e.g., lorlati). Note that by simplifying the factor Xa scaffold, both a higher score and higher quality of *de novo* compounds is achieved.

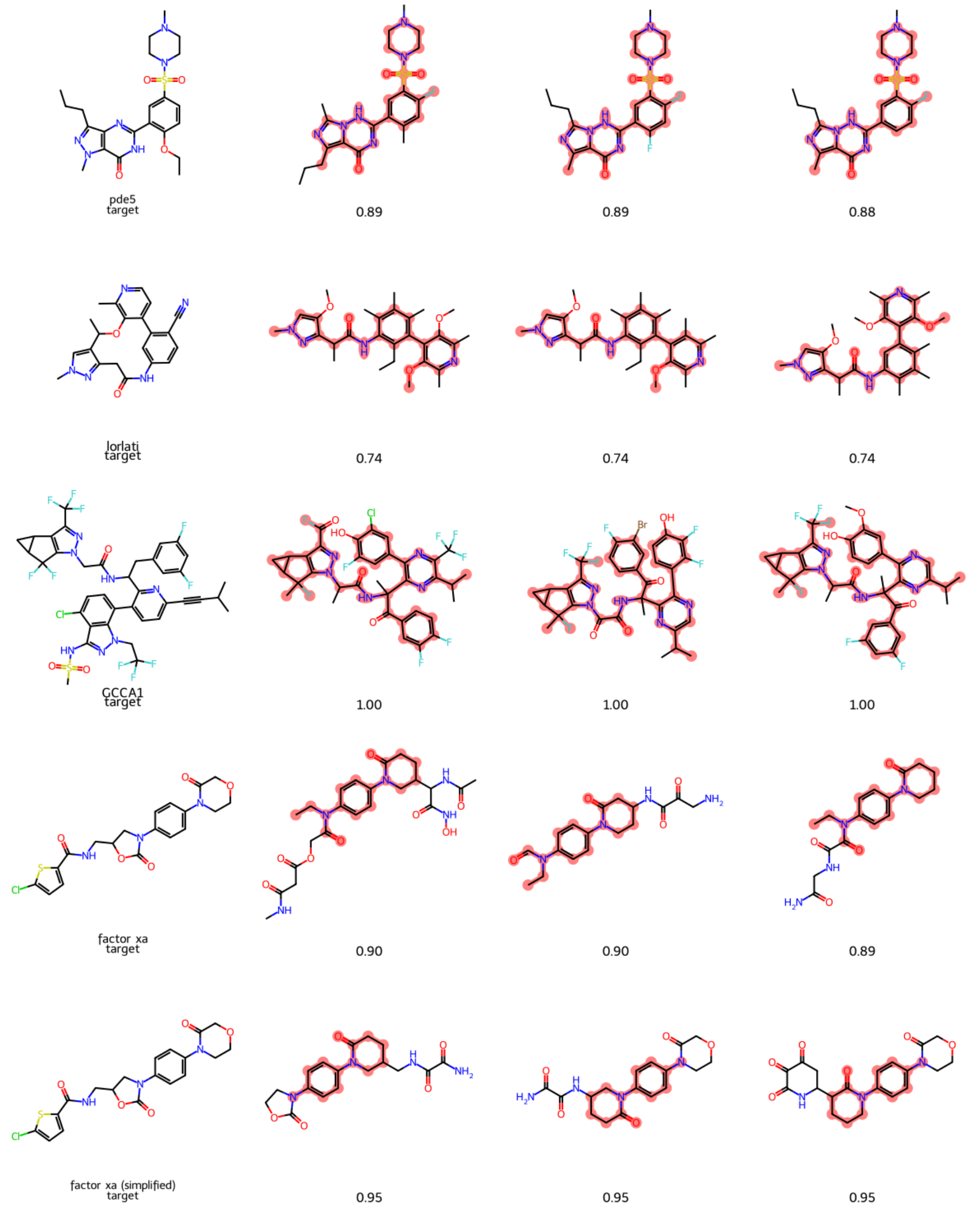

Fig. B8 Top 3 compounds proposed by PromptSMILES (optimise,shuffle) for each objective (row) in the GuacaMol Scaffolds benchmark. The chemical structure similarity target for each objective is shown in the first column and the *de novo* molecules in the right three columns. The scaffold used to seed scaffold decoration is highlighted in red. Note that it is not possible to achieve macrocyclisation for the lorlati objective (second row) via temporarily invalid SMILES strings, as our current implementation requires valid SMILES strings at each iteration.

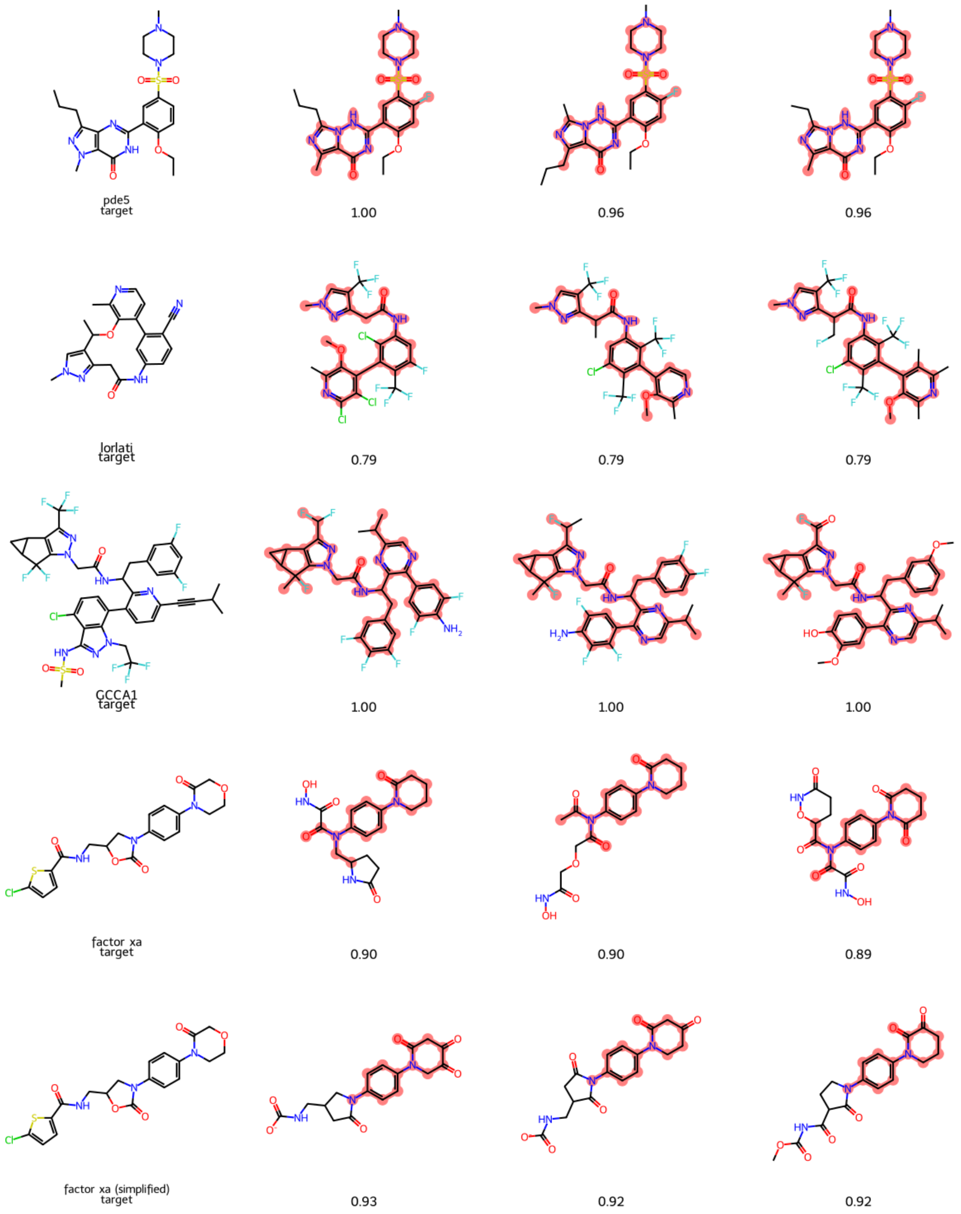

Fig. B9 Top 3 compounds proposed by PromptSMILES (optimise,shuffle,multi) for each objective (row) in the GuacaMol Scaffolds benchmark. The chemical structure similarity target for each objective is shown in the first column and the *de novo* molecules in the right three columns. The scaffold used to seed scaffold decoration is highlighted in red. Note that it is not possible to achieve macrocyclisation for the lorlati objective (second row) via temporarily invalid SMILES strings, as our current implementation requires valid SMILES strings at each iteration.

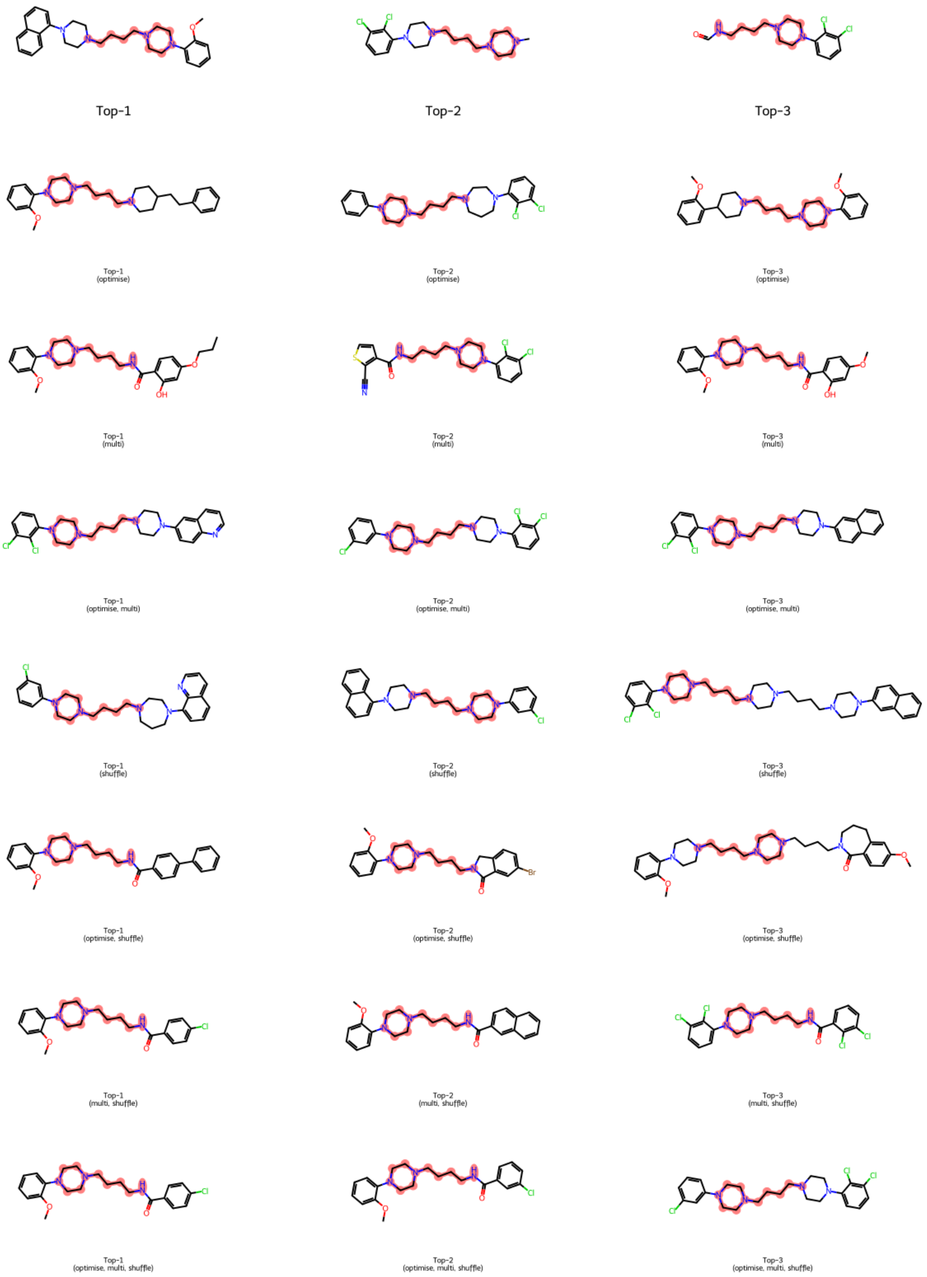

Fig. B10 Top 3 compounds proposed by PromptSMILES (with different parameters) for optimization of a QSAR model with no reaction filters as in LibINVENT with the seed scaffold highlighted in red.

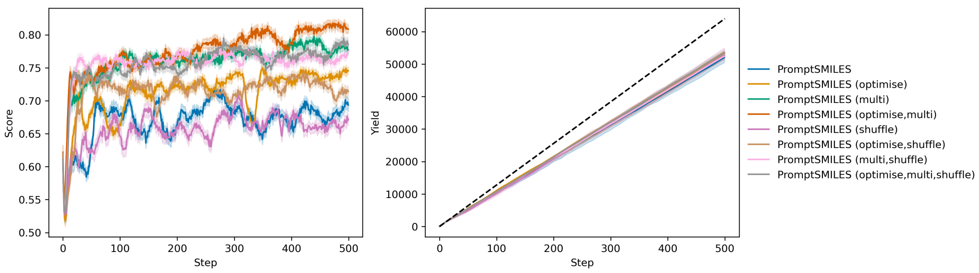

Fig. B11 Optimisation curve and yield of different PromptSMILES configurations for optimization of a QSAR model with no reaction filters as in LibINVENT. Experiments were replicated 3 times.

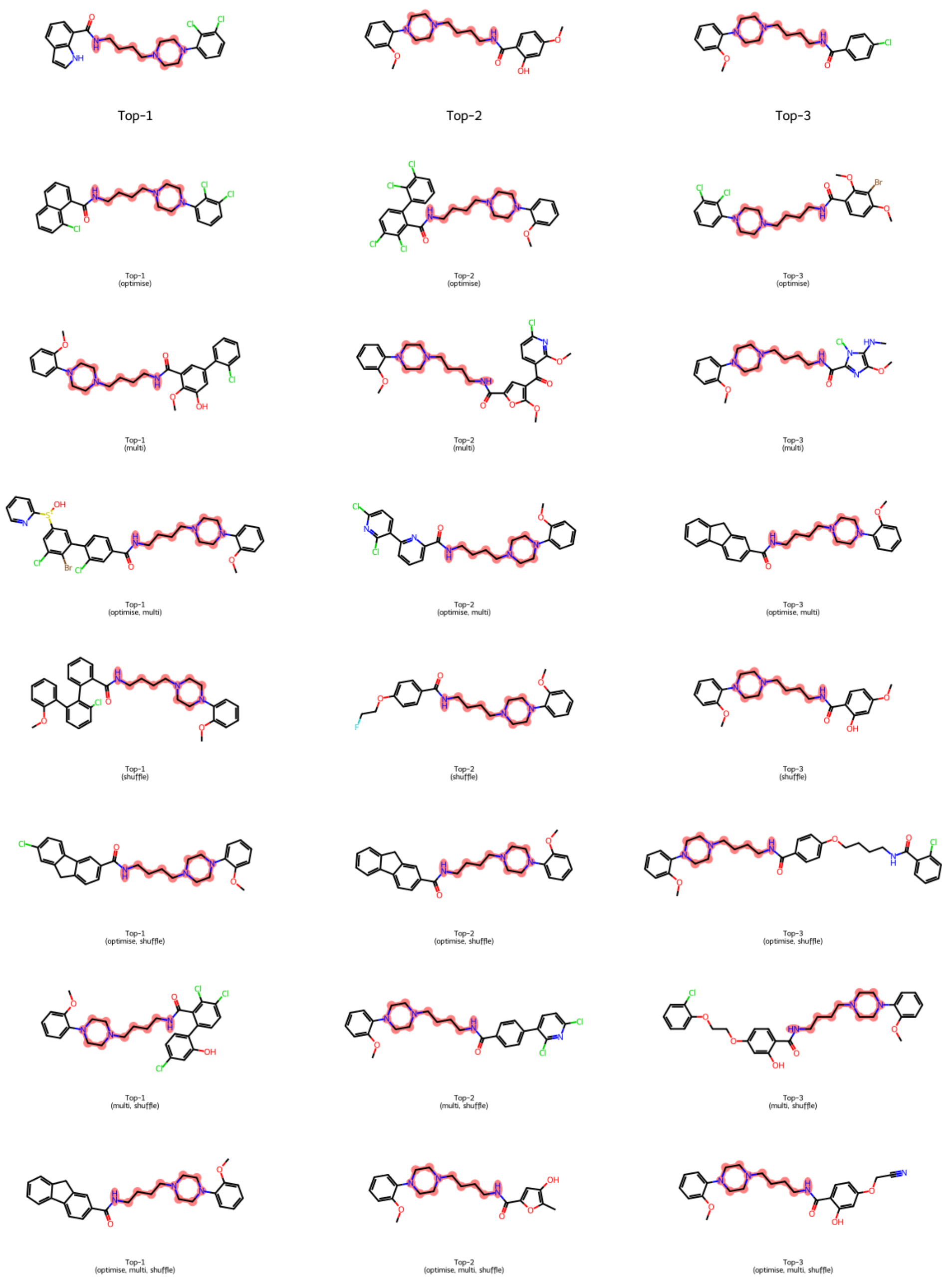

Fig. B12 Top 3 compounds proposed by PromptSMILES (with different parameters) for optimization of a QSAR model with selective reaction filters as in LibINVENT with the seed scaffold highlighted in red.

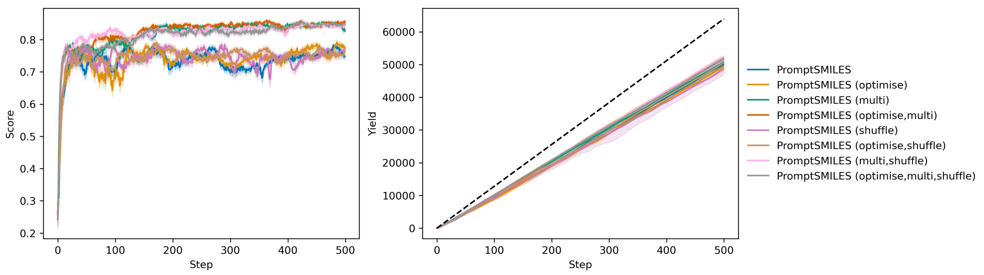

Fig. B13 Optimisation curve and yield of different PromptSMILES configurations for optimization of a QSAR model with selective reaction filters as in LibINVENT. Experiments were replicated 3 times.

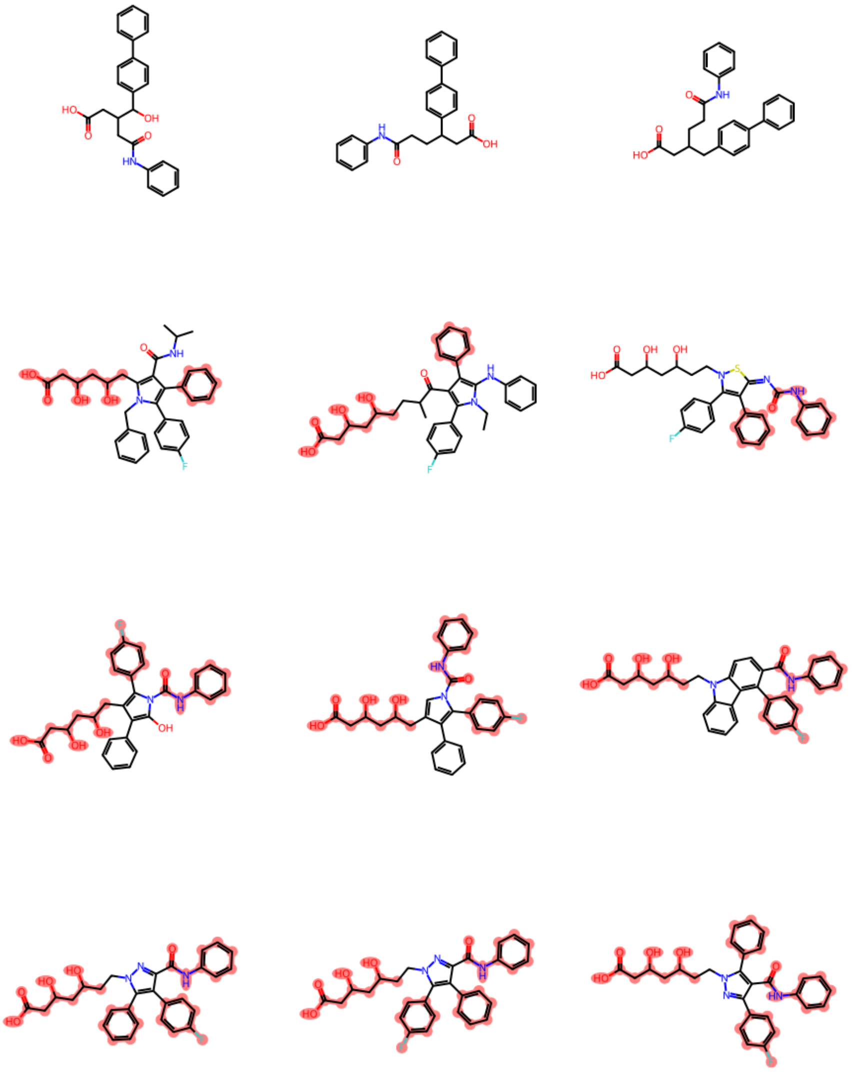

Fig. B14 Top 3 *de novo* compounds generated by PromptSMILES on similarity to Atorvastatin by number of seed fragments. Each row represents the top 3 compounds without using fragment linking (baseline), linking two fragments, linking three fragments or using four fragments respectively. Seed fragments used for a particular compound are highlighted.
